# Radiation therapy induces the DNA damage response in peripheral blood

**DOI:** 10.18632/oncotarget.1084

**Published:** 2013-06-26

**Authors:** Christopher J. Bakkenist, R. Kenneth Czambel, David A. Clump, Joel S. Greenberger, Jan H. Beumer, John C. Schmitz

**Affiliations:** ^1^ Department of Radiation Oncology, University of Pittsburgh School of Medicine, Pittsburgh, PA, USA; ^2^ Department of Pharmacology and Chemical Biology, University of Pittsburgh School of Medicine, Pittsburgh, PA, USA; ^3^ Molecular and Cellular Cancer Biology Program, University of Pittsburgh Cancer Institute, University of Pittsburgh, Pittsburgh, PA, USA; ^4^ Molecular Therapeutics and Drug Discovery Program, University of Pittsburgh Cancer Institute, University of Pittsburgh, Pittsburgh, PA, USA; ^5^ Lung and Thoracic Malignancies Program, University of Pittsburgh Cancer Institute, University of Pittsburgh, Pittsburgh, PA, USA; ^6^ Department of Pharmaceutical Sciences, University of Pittsburgh School of Pharmacy, Pittsburgh, PA, USA; ^7^ Division of Hematology Oncology, Department of Medicine, University of Pittsburgh School of Medicine, Pittsburgh, PA, USA

**Keywords:** DNA damage response, ATM

## Abstract

Stereotactic body radiation therapy (SBRT) is a radiotherapy modality that delivers highly conformal, ablative doses to a well-defined target. Here, using a semiquantitative multiplexed assay to analyze ATM and H2AX phosphorylation, we show that ATM kinase activity in peripheral blood mononuclear cells is induced following SBRT. This observation of a systemic ATM kinase-dependent DNA damage response in the peripheral blood is unprecedented and promotes the use of ATM serine-1981 phosphorylation as a predictive biomarker for DNA damaging modalities and ATM inhibitors.

## INTRODUCTION

Over half of the 1,638,910 Americans diagnosed with cancer in 2012 received radiotherapy. Stereotactic body radiation therapy (SBRT) is a treatment modality that relies upon target localization and motion compensation to deliver a conformal, ablative dose of radiation. While external beam radiotherapy is typically delivered in 25-40, 1-2 Gy fractions, 5 days per week, SBRT is delivered in 1-5, 8-36 Gy high dose fractions on nonconsecutive days. Although there is rapid dose fall-off from tumor into normal tissues, blood flow through the targeted volume is not restricted. We examined the induction of the DNA damage response in peripheral blood mononuclear cells (PBMCs) in blood drawn from patients before and after the first fraction of SBRT.

Since the majority of cancer therapies are DNA damaging, there is a pressing need for pharmacodynamic biomarkers of DNA damage. Histone H2AX serine-139 phosphorylation generates gamma-H2AX which was originally associated with the induction of DNA double strand breaks (DSBs)[1]. While it is now clear that gamma-H2AX is induced by agents that do not induce DSBs [2, 3], gamma-H2AX remains a widely used biomarker of DNA damage [4]. ATM is a kinase that initiates DNA damage signaling following exposure to ionizing radiation (IR) and other agents that induce DSBs. ATM kinase activity is associated with ATM serine-1981 phosphorylation in cells [5]. ATM serine-1981 phosphorylation is induced following 5 cGy [6] and facile extraction makes this phosphoprotein an excellent candidate biomarker.

## RESULTS AND DISCUSSION

**Figure 1:**
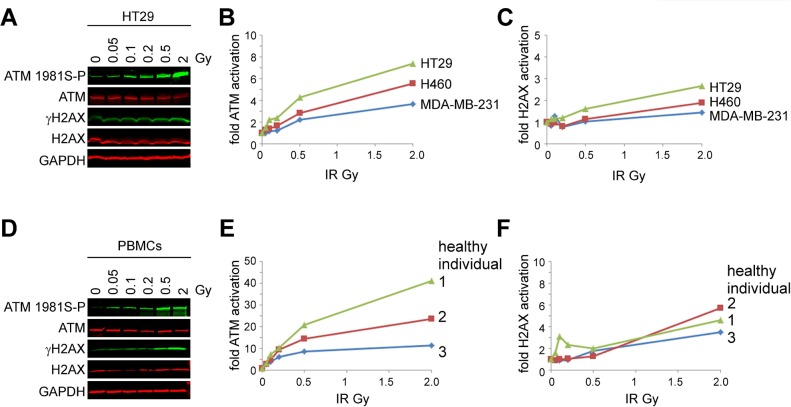
ATM serine-1981 phosphorylation induction by ionizing radiation is detected using a novel semiquantitative dual multiplexed immunoblot (A-C) Human cancer cell lines. (A) Pseudocolored images of the immunoblots generated from HT29 cells. (B) Ratio of ATM serine-1981 phosphorylation to total ATM protein in HT29 (colorectal), H460 (lung), and MDA-MB-231 (breast) cancer cell lines following exposure to IR. Ratios obtained from non-irradiated cells were normalized to 1. (C) Ratio of gamma-H2AX to total H2AX on the same immunoblot as in (B). (D-F) PBMCs. (D) Pseudocolored images of the immunoblots generated from PBMCs (healthy individual 1). (E) Ratio of ATM serine-1981 phosphorylation to total ATM protein in PBMCs following the exposure of whole blood to IR. (F) Ratio of gamma-H2AX to total H2AX on the same immunoblot as in (E).

To demonstrate the utility of ATM as a biomarker, we developed a semiquantitative multiplexed immunoblot to analyze ATM serine-1981 phosphorylation, total ATM protein, gamma-H2AX and H2AX. Human cancer cells were irradiated and whole cell extracts were resolved by SDS-polyacrylamide gel electrophoresis. Separate PVDF membranes were co-immunoprobed with anti-phosphoserine-1981 ATM and anti-pan-ATM antibodies as well as anti-gamma-H2AX and anti-H2AX antibodies. A dose-dependent induction of ATM serine-1981 phosphorylation was detected in the cancer cell lines HT29 (colorectal), H460 (lung), and MDA-MB-231 (breast), and this induction was greater than that of gamma-H2AX at each dose (Figure 1A-C).

To determine whether ATM serine-1981 phosphorylation is induced in PBMCs *ex vivo*, human blood was drawn from three healthy volunteers and exposed to IR. A dose-dependent induction of ATM serine-1981 phosphorylation was detected in PBMCs isolated from irradiated blood and this induction was greater than that of gamma-H2AX at each dose (Figure 1D-F). The dynamic range of ATM serine-1981 phosphorylation induction was 40-fold in PBMCs and 8-fold in cell lines suggesting that this response may be greater in cells that have not been cultured *in vitro*.

To test the hypothesis that ATM serine-1981 phosphorylation may be induced in the peripheral blood of SBRT patients, we analyzed ATM serine-1981 phosphorylation and gamma-H2AX in PBMCs isolated from blood drawn before and after the first fraction of SBRT.

**Figure 2:**
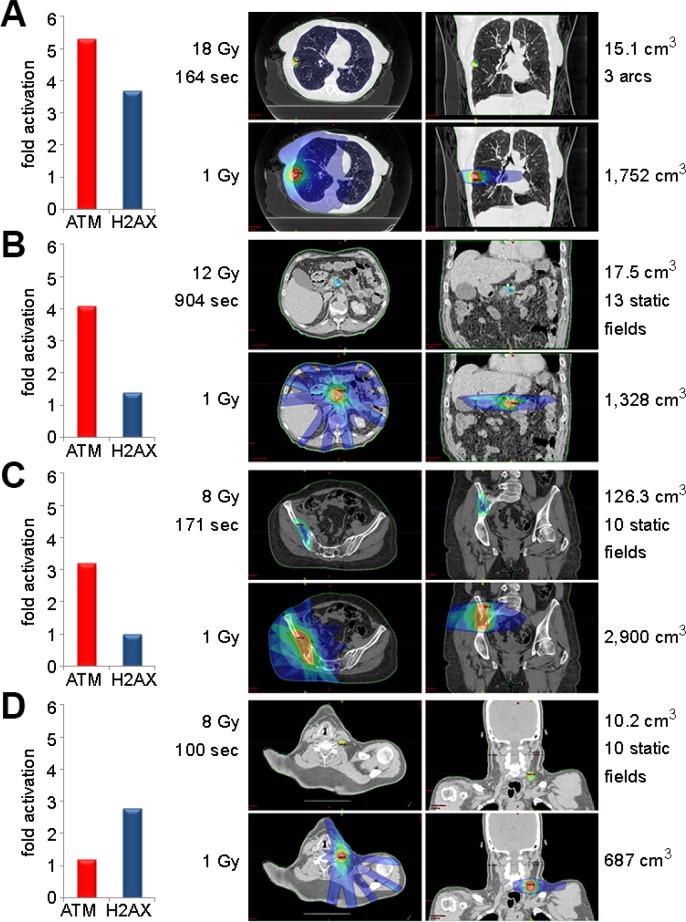
ATM serine-1981 phosphorylation is induced by SBRT (A) ATM serine-1981 phosphorylation and gamma-H2AX were induced 5.3-fold and 3.7-fold (left graph), respectively, in blood collected from a lung cancer patient before and after SBRT. Planning target volume (PTV) of 15.1 cm^3^ received a maximum of 20.0 Gy while the V1.0 of body was 9.1% (1,752 cm^3^). (B) ATM serine-1981 phosphorylation and gamma-H2AX were induced 4.1-fold and 1.4-fold, respectively, in blood drawn before and after SBRT for a pancreatic adenocarcinoma. PTV of 17.5 cm^3^ received a maximum dose of 13.93 Gy while the V1.0 of body was 7.9% (1,328 cm^3^). (C) ATM serine-1981 phosphorylation was induced 3.2-fold in blood collected before and after SBRT for a metastatic renal cell carcinoma with bone metastases. PTV of 126.3 cm^3^ received a maximum dose of 9.4 Gy while the V1.0 of the body was 10% (2,900 cm^3^). (D) ATM serine-1981 phosphorylation and gamma-H2AX were induced 1.2-fold and 2.8-fold, respectively, in blood drawn before and after SBRT for a metastatic cervical lymph node resulting from an esophageal adenocarcinoma. PTV of 10.2 cm^3^ received a maximum of 9.0 Gy while the V1.0 of body was 3.4% (687 cm^3^).

SBRT induced ATM serine-1981 phosphorylation and gamma-H2AX 5.3-fold and 3.7-fold, respectively, in a patient with inoperable non-small cell lung cancer (NSCLC) (Figure 2A). The prescribed planning target volume (PTV) was 15.1 cm^3^ with 18 Gy per fraction for a maximum of 20 Gy within the target volume and a beam-on time of 164 seconds. The maximum and mean dose to the heart was 2.0 and 1.0 Gy, respectively. Assuming the heart contains 280 cm^3^ of a total blood volume of 4,500 cm^3^, PBMCs within the heart of this patient received 6 cGy (280/4,500 × 1 Gy). This approximation is consistent with the 5 cGy that induces ATM serine-1981 phosphorylation in fibroblasts [6] and PBMCs (Figure 1E).

SBRT induced ATM serine-1981 phosphorylation and gamma-H2AX 4.1-fold and 1.4-fold, respectively, in a patient treated following pancreatectomy for pancreatic adenocarcinoma (Figure 2B). The prescribed PTV was 17.5 cm^3^ with 12.0 Gy per fraction for a maximum of 13.9 Gy within the target volume and a beam-on time of 904 seconds.

SBRT induced ATM serine-1981 phosphorylation 3.2-fold in a patient with metastatic renal cell carcinoma with bone metastases (Figure 2C). No induction of gamma-H2AX was observed. The prescribed PTV was 126.3 cm^3^ with 8.0 Gy per fraction for a maximum of 9.4 Gy within the target volume and a beam-on time of 171 seconds.

SBRT induced ATM serine-1981 phosphorylation and gamma-H2AX 1.2-fold and 2.8-fold, respectively, in a patient treated for adenocarcinoma of the gastroesophageal junction post esophagectomy (Figure 2D). The prescribed PTV was 10.2 cm^3^ with 8.0 Gy per fraction for a maximum of 9.0 Gy within the target volume and a beam-on time of 100 seconds.

To our knowledge this is the first documentation of ATM serine-1981 phosphorylation in patients following radiotherapy. ATM serine-1981 phosphorylation has unequaled sensitivity for the detection of DNA damage in cells cultured *in vitro* [5, 6] and in human tissues [7]. The data presented here show that ATM serine-1981 phosphorylation has an even greater dynamic range in PBMCs irradiated *ex vivo*. ATM serine-1981 phosphorylation may be an excellent biomarker for exposure to IR and other DNA damaging agents in human patients and as such may have utility in predicting therapeutic response and as measure of radiation exposure in counter-terrorism efforts.

Finally, it has long been postulated that systemic ATM kinase inhibition may be an excellent means to increase the efficacy of targeted radiotherapy [8, 9]. Our data show, for the first time, that even the most guided radiation therapy is associated with a systemic DNA damage response. Therefore, systemic ATM kinase inhibition may be associated with DNA damage injury in circulating PBMCs. In this regard the absence of detectable ATR expression in PBMCs [10] is interesting because it suggests that the biological responses to DNA damage in PBMCs may be strictly dependent on ATM and as such ATR kinase inhibition may be a better means to selectively increase the efficacy of targeted radiotherapy. Our observation of a systemic ATM kinase-dependent DNA damage response through the peripheral blood following SBRT is unparalleled and promotes the use of ATM serine-1981 phosphorylation in evaluating the biological effects of DNA damaging modalities and ATM inhibitors.

## MATERIALS AND METHODS

### Materials

Anti-histone H2AX mouse IgG2A monoclonal antibody (clone 322105) was purchased from R&D Systems, Inc. (Minneapolis, MN). Biotinylated anti-phosphoserine-139 histone H2AX rabbit monoclonal antibody (clone 20E3) was purchased from Cell Signaling Technology, Inc. (Danvers, MA). Anti-pan-ATM purified mouse monoclonal antibody (clone MAT3-4G10/8) was purchased from Sigma-Aldrich (St. Louis, MO). Anti-human phosphoserine-1981 ATM rabbit monoclonal antibody (clone EP1890Y) was purchased from Epitomics (Burlingame, CA). Anti-GAPDH mouse monoclonal antibody (clone O411) was purchased from Santa Cruz Biotechnology (Dallas, TX). Odyssey® blocking buffer, IRDye 800CW conjugated goat anti-rabbit IgG (H + L), and IRDye 680RD conjugated goat anti-mouse IgG (H + L) antibodies were purchased from LI-COR Biotechnology-US (Lincoln, NE). DyLight 800 conjugated streptavidin was purchased from Thermo Fisher Scientific, Inc. (Rockford, IL). Glycine, Tween 20, Tris, 10x Tris/Glycine/SDS electrophoresis running buffer, precision plus protein standards (10 – 250 kDa), Tris-HCL and 4-15% TGX Criterion™ precast (18-well) gels were purchased from Bio-Rad Laboratories (Hercules, CA). Phosphatase and protease inhibitor cocktail tablets were purchased from Roche Diagnostics (Indianapolis, IN). Plasma-Lyte A USP was purchased from Baxter (Deerfield, IL). Any other chemicals were of analytical grade and purchased from Sigma-Aldrich Corp. (St Louis, MO).

### Cell culture and irradiation

HT29, MDA-MB-231, H460 cells were purchased from the American Type Culture Collection (ATCC). Cells were cultured in RPMI-1640 (Life Technologies) with 10% fetal bovine serum (Gemini Bio-Products). Cells were tested monthly for mycoplasma by the MycoAlert Mycoplasma detection assay (Cambrex BioScience; Rockland, ME). Cells were gamma-irradiated in a Shepherd Mark I Model 68 [^137^Cs] irradiator (J.L. Shepherd & Associates) at a dose rate of 71.1 Rad/min.

### Blood Collection and PBMC Isolation

Human blood (~8.0 mL/tube) was drawn into Cell Preparation Tubes (CPT) (Becton Dickinson, Franklin Lakes, NJ) during a single venipuncture event in accordance with an Institutional Review Board (IRB) protocols (#PRO13020420, #PRO10020482, #PRO10050157, #PRO11010576). PBMCs were isolated by centrifugation at 1500 × g for 30 min at room temperature (RT). The PBMC layer was transferred to a new 15 mL polypropylene conical tube and washed with 3-4 volumes of Plasma-Lyte A USP. The cells were pelleted by centrifugation (430 × g for 10 min at RT). The PBMCs were resuspended in 3.0 mL of Plasma-Lyte A. Immediately, 20 microliters of the cell suspension was removed and used to estimate PBMC density with a Beckman Coulter Z1 particle counter before an additional wash of 10 mL in Plasma-Lyte A. After re-pelleting, aspiration and disposal of the supernatant, the PBMC density was adjusted to 3 × 10^6^ cells/mL in Plasma-Lyte A. Single-use aliquots of 0.5 mL were then transferred into new 2.0 mL micro-tubes. Lastly, the PBMCs were recovered as cell pellets by centrifugation (10,000 × g for one min at RT), and flash-frozen in a dry ice/ethanol bath before storage at −70°C until use. To preserve specimen integrity, all CPT tubes were processed within three hours of collection.

### Whole Cell Extract Preparation

Frozen cell pellets were resuspended in DTT-modified Laemmli buffer (50 mM Tris-HCl (pH 6.8) containing 2% (w/v) SDS, 10% (v/v) glycerol, 200 mM DTT and 0.002% (w/v) bromophenol blue) freshly supplemented with protease and phosphatase inhibitor cocktails, vortexed and pulsed sonicated in an ice-cold cup-horn sonicator (Cole-Parmer, Vernon Hills, IL). Lysates were heated to 95°C for 7 min, vortexed and chilled briefly on ice. After centrifugation at 16,000 × g for 5 min at RT, supernatants were transferred into new 1.5 mL micro-tubes.

### Detailed Immunoblot Procedure

To avoid bias in protein levels due to possible plasma protein contamination, PBMC lysates were normalized by cell number [11]. Aliquots of 25 microliters (5 × 10^5^ cells) were loaded per well onto 4-15% TGX Criterion™ 18-well precast gels in a Criterion™ vertical midi-format electrophoresis cell (Bio-Rad Laboratories, Hercules, CA). Proteins were fractionated under a constant 200 V applied for ~40 min. Resolved proteins were electrophoretically transferred onto 0.45 micrometer Immobilon-FL polyvinylidene difluoride (PVDF) transfer membranes (Millipore, Billerica, MA) with 100 V applied for 1.5 h in a Criterion blotter transfer tank (Bio-Rad Laboratories, Hercules, CA). Following electroelution transfer, membranes were rinsed with water and incubated in 0.5x Odyssey® blocking buffer (20 mL/membrane) for 1 h at RT with gentle agitation. The membranes were cut horizontally into three strips. The top membrane strip (above 75 kDa) was co-immunoprobed with an anti-phosphoserine-1981 ATM and anti-pan-ATM antisera mixture diluted to 1:500 and 1:2500, respectively, in 0.5x Odyssey® blocking buffer containing 0.1% Tween 20 (OBB-T). The middle membrane (25-75 kDa) was immunoprobed with anti-GAPDH diluted 1:20,000 in OBB-T. Likewise, the bottom membrane strip (below 25 kDa) was simultaneously probed with a biotinylated monoclonal rabbit anti-gamma-H2AX antibody and a mouse monoclonal anti-H2AX antibody, each diluted to 1:1000 in OBB-T. Membranes were incubated overnight at 4°C with gentle agitation. After removing the antisera, the membranes were rinsed with TBS, washed thrice with 50 mL of TBS-T, for periods of 10 min each. The ATM membrane was immunoprobed with highly cross-absorbed anti-IgG detection antibodies that were individually matched to the specific host species in which the primary ATM antibodies were generated. The individual detection antisera mixture antibodies, a goat anti-rabbit IgG and a goat anti-mouse IgG antibody were labeled with NIR fluorochrome dyes (800 and 700 nm) and diluted 1:4000 and 1:5000, respectively. The H2AX membrane was similarly probed except both antisera in the mix were diluted 1:5000 and in addition, the mixture included streptavidin conjugated to an 800 nm fluorophore diluted 1:10000. All detection reagents were prepared in OBB-T containing 0.02% (w/v) SDS. The strips were incubated in the dark for 1 h at RT with gentle agitation. After incubating, and washing thrice for 5 min each in 50 mL of TBS-T, to remove Tween-20, membranes were washed twice more for 10 min in 50 mL of TBS. All five washes were carried out undulating in the dark at RT. Before proceeding, membranes were dried at RT for at least 1 h in the dark.

### Immunoblot Imaging and Signal Quantification

Dry membrane strips were visualized using an Odyssey® CLx infrared imaging system (model 9120). Signal quantification was performed utilizing the version 2.1 Image Studio software (LI-COR Biosciences-US, Lincoln, NE). Shapes were drawn around bands of interest, and the signal was calculated as the sum of the individual pixel intensity values (Total) for the selected shape minus the product of the average intensity values of the pixels in the background (Bkg) and the total number of pixels enclosed by the shape (Area). Thus, Signal = Total – (Bkg × Area). The selected background value applied for each individual shape was the median of three pixels above and below the shape. The ratio of the phosphorylated to pan-protein signal was used to correct for loading variations between lanes.
